# A Virtual Approach to Promote Inter-Professional Learning (IPL) Between Biomedical Science and Medicine in Higher Education for the Benefit of Patient Care

**DOI:** 10.3389/fpubh.2021.747751

**Published:** 2021-10-06

**Authors:** Wendy Leadbeater, Ross Pallett, Elizabeth Dunn, Amreen Bashir

**Affiliations:** ^1^Aston Medical School, College of Health and Life Sciences, Aston University, Birmingham, United Kingdom; ^2^Aston Pharmacy School, College of Health and Life Sciences, Aston University, Birmingham, United Kingdom; ^3^School of Biosciences, College of Health and Life Sciences, Aston University, Birmingham, United Kingdom

**Keywords:** interprofessional education (IPE), interprofessional learning, biomedical science, medicine, healthcare professions, virtual education, cross-discipline collaboration

## Abstract

In the clinical setting, collaboration between multidisciplinary teams is core to providing effective patient care. The delivery of traditional interprofessional education is associated with a number of logistical challenges, which were heightened by the Covid-19 pandemic. This workshop was developed to bring together Biomedical Science and Medical students using an online platform. The workshop consisted of (1) defining interprofessional education, (2) introducing the role of the Pathology laboratory, (3) Professional registration with regulatory bodies and (4) an insight into Covid-19 laboratory diagnosis. The session was supported by mixed group breakout rooms and interactive polling. Thirty four percent of students completed a post-workshop online survey which included open and closed questions. Thematic analysis revealed a better understanding the role of the pathology laboratory in diagnosing disease, an increased awareness of the similarities and differences in the roles of a Biomedical Scientist and a Medic and the importance of a multi-disciplinary team in achieving effective patient care. Quantitative analysis of survey data revealed that the majority of students reported positive experiences of interprofessional education online. Approximately 90% of students agreed that the workshop enabled them to increase their understanding of their own roles within healthcare, in addition to increasing their understanding of the roles of other healthcare professionals. 74.3% of participants reported that working with students from a different programme provided an alternative perspective. Seventy nine percent of students agreed that the online format enabled interactivity and discussion of the tasks. Of the 204 students, 85% engaged with the four polls during the workshop. This online workshop enabled discussion between degree programmes, enabled interactivity and allowed the learning outcomes to be met. Universities should embrace online platforms to provide a novel, engaging and effective interprofessional educational experience.

## Introduction

In the clinical setting, the importance of interprofessional approaches to education and practise has been highlighted, ranging from improving the quality of patient care and safety, to reducing clinical errors (1, 2). Modern patient care is reliant upon healthcare professionals from different disciplines working collaboratively. This includes understanding each other's roles and contributions to the multi-professional team using their skillsets to problem-solve and interpret medical jargon for effective patient care (1). Interprofessional Learning (IPL) within Higher Education (HE) aims to bring students enrolled on two or more undergraduate degree programmes, to learn with, from and about each other, with the goal of achieving common learning outcomes (3, 4). Together they learn about each other's roles, skills and responsibilities, and the value of effective communication across disciplines (5), in preparation for clinical practise. The World Health Organisation (WHO) recognised the importance of IPL in 1978 and highlighted if health professionals were taught collaboratively in a multi-professional education setting, they were more likely to work effectively together in the clinical setting (6).

## Recognition of IPL by Regulatory Bodies

IPL is recognised by the Professional Statutory and Regulatory Bodies (PSRBs). The HE curriculum is underpinned by standards set by PSRBs. The General Medical Council (GMC) sets the standards and requirements for delivery of medical education, and medical school programmes must provide medical students opportunities to work and learn from students of other professional programmes to support multi-disciplinary working (7). The Health Care and Professions Council (HCPC) accredit allied healthcare professional courses within the UK and recognise the importance of Biomedical Scientists working with, and from learners of other relevant professions (8). To prepare more inclusive and dynamic healthcare teams, an understanding of and opportunities to undertake IPL is essential. This will help overcome barriers such as a lack of awareness of the roles within the healthcare system and their involvement in patient care (9, 10).

## Challenges in the Implementation of IPL

In HE, there are often logistical challenges that accompany IPL due to the large number of students (face to face learning) involved in traditional IPL, these include timetabling across multiple disciplines, as well as constraints on suitable learning environments and space (11). Another challenge that universities face when delivering IPL is to ensure that the activities are relevant to all programmes involved and activities are embedded into the curriculum, rather than extra-curricular activities which typically garner the interest of only a small proportion of students (4).

## Opportunities Provided by the Use of an Online Platform

In HE, the move to online learning platforms provides a solution to overcome these challenges and delivers an authentic opportunity for students to work together using collaborative practise and problem based learning (PBL). This creates a different learning environment and alternative approach to working in interprofessional groups. PBL has been a key feature of medicine and allied health care programmes for a number of years (12, 13). A recent study demonstrated additional value of co-constructive discussions between students from different backgrounds, with the common goal of addressing patient concerns (1).

The Covid-19 pandemic and the extraordinary stresses it placed on the National Health Service (NHS) highlighted the importance of effective interprofessional working relationships in order to collaboratively overcome the many urgent, complex and unique demands that arose (14). IPL has been trialled virtually between healthcare focused courses and studies have reported improved teamwork, interprofessional knowledge and attitudes (15–17). Whilst a number of studies have evaluated the effectiveness of IPL programmes delivered through traditional on-campus workshops, there are relatively few studies which assess the delivery of IPL using online virtual platforms. Due to the impact of Covid-19 most UK universities moved to online delivery of learning and teaching to protect both staff and students. The aim of this study was to evaluate the effectiveness of an IPL activity designed to improve awareness and understanding of the roles of Biomedical Scientists (BMS) and Medics within healthcare, using PBL and an online platform for delivery.

## Materials and Methods

### Creation of an Online IPL Workshop

The IPL workshop was created and co-delivered by academics from the School of Biosciences, the Medical School and Pharmacy School, Aston University, UK. The workshop was scheduled for 3 h and consisted of academic-led material and breakout rooms using the Blackboard Collaborate platform (Blackboard, Washington DC). Taught topics were delivered to the entire student cohort of final year Biomedical Science and second year medical students and included (1) defining IPL, (2) introducing the role of the Pathology laboratory, (3) Professional registration with regulatory bodies and (4) an insight into Covid-19 laboratory diagnosis. The workshop included a series of breakout sessions where students worked in small mixed discipline groups (6 students per group) for 20 min to complete three activities. In total there were 34 breakout rooms, which were supervised by 4 members of staff. At the end of each breakout session, students were brought back to the main virtual room to discuss the activity and share answers from each perspective. Students were able to interact with academics throughout the entire workshop using the chat function. An activity booklet was designed and completed by one member of each group, prior to being uploaded onto the virtual learning environment (VLE). The five page booklet was designed to motivate students to participate within the study and to promote engagement with the activities within the breakout rooms. Students completed three activities which required input from both Biomedical Science and Medical students.

### Activities in the Breakout Groups Included

1. Assign key skills and roles to the following healthcare professions: Medical Doctor, BMS and a Pharmacist.
2. Explain the purpose of a range of different blood tests along with clinical investigations e.g., measuring blood pressure and heart rate.
3. Share how Medical Doctors and BMS obtain registration with their professional body.

Four interactive polls were created based upon given scenarios presented throughout the workshop. The polls were hosted using the Blackboard Collaborate platform and assessed students application of knowledge and their understanding of processes related to patient sample collection, processing and diagnosis of disease.

### Collecting Student Feedback and Analysis of Results

Final year Biomedical Science and second year Medical student experiences of IPL using an online platform were collected post-workshop through an eleven-item online questionnaire. A mixed methodology approach was adopted, which included open and close ended questions. The results were analysed both quantitatively and qualitatively. Students were invited to participate in the study during the IPL session and were provided the link to an online survey on Blackboard and through the chat function. Explanatory statement and consent was embedded within the online survey. Consent was required prior to accessing the questions. Students completed questions measuring awareness of professional roles, learning with and from one another (collaboration and knowledge gain), the role of IPL in patient care and the effectiveness of the online workshop (structure and organisation) in creating an IPL environment (Table 1). Questions around teamwork, roles and collaboration were influenced by Parsell and Bligh and were developed based on the expert opinion of the investigators (18).

**Table 1:** Student survey responses relating to the IPL workshop (*n* = 70).

**Statements**	**Biomedical Science**	**Medicine**	***P***	**Percentage “Agreed” or “Strongly Agreed”**
	**(mean ± SD)**	**(mean ± SD)**		
1. Working with students and staff from other programmes improved my awareness of the role of other professionals, in the patient pathway	4.21 ± 0.90	4.55 ± 0.57	0.09	90%
2. Working with students from other professional programmes will help me understand my own (professional) role and limitations	4.22 ± 0.85	4.38 ± 0.86	0.44	88.5%
3. I need to know about the role of other professionals for my future practise	4.39 ± 0.74	4.69 ± 0.47	0.06	94.3%
4. I feel the online platform allowed me to meet the learning outcomes of the session	3.98 ± 1.06	4.10 ± 0.98	0.61	78.5%
5. Working with students from a different programme provided an alternative perspective (which may not have been considered if working with own programme alone)	3.93 ± 1.17	4.17 ± 1.07	0.37	74.3%
6. Learning with, from and about each other (from different programmes) will improve my team work skills and working relationships	4.39 ± 0.80	4.57 ± 0.50	0.29	95.6%
7. Patients will ultimately benefit if professionals work together and learn from each other	4.73 ± 0.45	4.79 ± 0.41	0.56	100%
8. I enjoyed working with students on the other programme on this activity	4.07 ± 1.23	4.31 ± 0.93	0.39	82.9%
9. I have a better understanding of the other students role, from their contribution to the scenario discussed	3.88 ± 1.14	4.45 ± 0.78	0.02*	80%
10. The session enabled discussion between the two programmes involved	4.12 ± 1.19	4.31 ± 0.93	0.48	84.3%
11. The online format enabled interactivity and discussion of the tasks/scenario presented	4.02 ± 1.19	3.97 ± 1.08	0.83	78.6%

The mean Likert scores for each statement are provided using a five point Likert scale with 1 = strongly disagree and 5 = strongly agree ± the standard deviation (SD). A two-tailed t-test was used to determine significance (^*^p < 0.05) between the Biomedical Science and Medical student cohorts. The bold value demonstrates a statistically significant value.

Answers included Likert-scales, free-text options and interactive polling. The study used the Online Surveys (formerly known as Bristol Online Survey; BOS) platform (JISC, Bristol, UK). Participation was anonymous and voluntary; no demographic was collated apart from the degree programme on which the students were enrolled. The study was approved by the Life and Health Sciences Research Ethics Committee (Project #1494). The survey was available for 2 weeks (17th December 2020–31st December 2020). The survey was advertised during the IPL session and using the Blackboard announcement feature which included links to the online surveys and reminders sent via announcements and email.

To account for variations in the number of responses to a given question, the number of respondents to each question has been specified. We have included the raw data (where appropriate) as the study population was small, so there is not always statistical significance. To compare responses between Biomedical Science and Medical students, unpaired two-tailed *t*-tests were used to determine statistical significance between the two student cohorts (*p* < 0.05).

The free-text responses were analysed using Thematic Analysis (19). The procedure was as follows: the researchers individually read the data multiple times for familiarity and generated initial codes; the codes were then collated to form initial themes; the themes were then reviewed against the data set for plausibility; and finally the themes were refined. This process was repeated by two members of the research team individually for triangulation and final themes were agreed collectively to produce the thematic analysis.

## Results

A total of 204 students engaged with the workshop, of which 70 students (34% response rate) completed the online questionnaire, these consisted of 59% Biomedical Science students and 41% Medical students. Participants responded to a series of statements related to the importance of interprofessional education and its role in the workplace. Table 1 shows that both student cohorts responded positively to the statements relating to the workshop: overall, 73% of Biomedical Science students and 91% of Medical students stated that they “Agree” or “Strongly Agree” with the statements.

All statements were rated highly across both cohorts, for all measures. Students “Agreed” or “Strongly agreed” they had improved awareness of the role of the other profession, their own role and limitations, with the understanding they need to know the roles of other healthcare professions in the team for future practise (statements 1, 2, 3). Within the knowledge measure, 80% of students across both cohorts “Agreed” or “Strongly agreed” that they now have a better understanding of the other students role as a result of this workshop, in particular medical students significantly (*p* < 0.05) improved their understanding of Biomedical Science students role due to the scenario discussed (statement 9). Students had an increased appreciation and alternative perspective by working with students from a different programme (statement 5). In particular, all students “Agreed” or “Strongly agreed” that collaboration across professions will benefit patients (statement 7). Where the workshop enabled discussion between the two programmes and improved their team work skills and relationships (statements 6, 10). The students from both cohorts “Agreed” or “Strongly agreed” that the structure and organisation of the session (statements, 4, 8, 11) meant they enjoyed the activity and felt the online platform enabled them to meet the learning outcomes and supported interactivity and discussion.

Prior to the IPL workshop, all students completed a Haematology module in which sample collection and pre-analytical processing was taught. An understanding of these processes were assessed using interactive polling, of which Table 2 shows the results.

**Table 2:** Responses to interactive polling.

**Poll question**	**Student response rate**	**Number of correct responses**
Q1. Which of the following shows the correct order of draw?	172/203 (85%)	70 (41%)
Q2. Which of the following statements are correct about serum?	173/204 (85%)	97 (56%)
Q3. Which two tests are performed to diagnose Covid-19?	160/197 (81%)	79 (40%)
Q4. Which antibody is a marker of past infection?	165/196 (84%)	120 (61%)

All polling questions asked within the workshop had high response rates from 81 to 85%. The questions were anonymous, therefore indication of student number responding from each programme could not be identified. Question 1 and 2 focused upon sample collection and the preparation for testing, covered prior to the workshop. Question 1 demonstrated that 41% of respondents identified the correct order of draw, and Question 2 showed that 56% identified the correct statements relating to serum. These polling results for Question 1 and 2 illustrate the need for this content to be reinforced across both student cohorts and that incorrect sample collection and preparation prior to testing can negatively impact patient care.

Within the workshop both student cohorts were taught new information about diagnostic tests used to screen for Covid-19. For Question 3, only 40% of students correctly identified the two tests used in the laboratory to diagnose coronavirus infection. The final polling question (Question 4) asked students to identify which antibody is a marker of past infection, knowledge which is relevant for both student cohorts and is taught as part of their degree programme. As shown in Table 2, 61% of students identified the correct answer.

### Free Text Responses: Thematic Analysis

To gauge a better understanding of what students learned from and taught each other, a thematic analysis was conducted of the responses to the free-text questions: Q8 - *What did you learn from the other students on the programme?* and Q9 - *What did you feel you taught your peers when sharing your ideas and experience?* (Appendix 1 in Supplementary Material). In total, 80% (*n* = 56) of the students answered question 8, whilst 73% (*n* = 51) answered question 9. Responses were analysed and common themes were identified between the two questions. The three most prominent themes identified were as follows:

1. The role of a Pathology laboratory in diagnosing disease,
2. An understanding of the similarities and differences in the roles of a BMS and Medic
3. Importance of a multi-disciplinary team in achieving effective patient care.

### Theme 1: The Role of the Pathology Laboratory in Diagnosing Disease

Six medical students reported that they learned of the importance of the Pathology laboratory in diagnosing disease and the majority of the responses indicated that they learned about different tests that are performed on patient samples within a diagnostic laboratory. Comments included:

**“*The fact that they have the same goal as us medical students which is to improve patient's health but they do different tasks such as laboratory investigations on samples which is not done by doctors.”***

**“*I learned about different tests that are performed in the lab to diagnose sepsis and to identify the key markers that would be out of range from lab results.”***

Similarly, ten Biomedical Science students reported that they taught the other student cohort about the role of the Pathology laboratory in diagnosing disease during the workshop. Specific comments related to the diagnostic tests and the need for medics to correctly fill out laboratory request forms in order for Biomedical Scientist's to process the samples. These results are then released to the Medic to appropriately treat the patient. Comments included:

**“*I taught other students the purpose of the diagnostic tests and how the BMS laboratory works, along with the importance of Medics correctly filling out request forms.”***

**“*My role in the testing process and the type of work undertaken by a BMS; I shared how to interpret a full blood count and stressed that this test does not only look for anaemia, but also diagnose infection through checking the white cell count.”***

### Theme 2: An Understanding of the Similarities and Differences in Each Other'S Career Pathways

Twenty nine students reported that they gained an understanding of the roles of a Biomedical Scientist and a Medic. The majority of the responses indicated that students gained a deeper insight into the roles that each professional undertakes and learned how their roles are linked. Comments included:

**“*A deeper understanding of the role of a Biomedical Scientist.”***

**“*A clear understanding of the role of a Biomedical Scientist and how it overlaps with doctors and pharmacists.”***

**“*The similarities and differences in some of the tasks performed and how their role is linked to ours.”***

**“*The “behind the scenes” whenever a doctor requests a test or prescribes drugs.”***

Furthermore, students also learned about differences and similarities in professional registration and how to legally practise their profession in the UK. Comments on what they learned included:

**“*The steps that are required to register as a Medic and the different responsibilities associated with their roles.”***

**“*The route to become a registered BMS I was not previously aware of.”***

Thirty one students reported that they taught the other student cohort about their role in the patient pathway. Various students reported that they gave insights into their role and the importance of their jobs in effectively treating patients. Furthermore, students also appreciated the roles of other professionals involved in the patient pathway. Comments included:

**“*I believe I gave Medics an insight into what a BMS is and what they do and how they are involved in patient treatment.”***

**“*I taught medics the role a BMS has in interpreting and relaying results from tests requested.”***

**“*I taught the BMS students the roles of a doctor that they may not have already been aware of and that the doctor is the centre piece connecting these specialities to patient care.”***

**“*I taught BMS the different roles of a doctor - not just talking to patients and doing a few tasks. The appreciation medical students have for other professionals (often understated).”***

Again, many students reported that they taught the other cohort how to become a registered professional in the UK and the different accrediting bodies that are involved. Examples of statements included:

**“*More about the role of a medical doctor and how to become a registered doctor.”***

**“*Our route to practise as a registered Biomedical Scientist and the importance of the HCPC in gaining registration in order to practise.”***

### Theme 3: Importance of a Multi-Disciplinary Team in Achieving Effective Patient Care

Nine students reported that through the workshop they gained an appreciation of how working with other health professionals can deliver effective patient care. Students reported that they understood the importance of working collaboratively and how each professional plays an important role in treating the patient. Comments included:

**“*It allowed me to understand how the two programmes are linked and their importance when working together.”***

**“*Learnt more about the roles and responsibilities of medics and where BMS can interact with them and work together to help diagnose patients.”***

**“*The importance of effective communication between Medics and BMS.”***

Similarly, eight students reported that they taught the other cohort the importance of working together, stressing the need for a collaborative approach to provide high quality patient care. Comments included:

**“*How many overlaps there are in our roles and how we have to work collaboratively to benefit the patient.”***

**“*The workshop allowed discussions about how we interact with different professionals and I stressed how we need to work together to interpret results and what they mean.”***

**“*We were able to work great as a team and listened to each other and respected each other opinions which is what we need to do when working in a hospital.”***

## Discussion

This study sought to determine the effectiveness of delivering IPL online using PBL, between Biomedical Science and Medical students. The workshop required students to collaboratively work together across degree programmes and aimed to increase awareness and understanding across professions.

### The Role of IPL in Healthcare Orientated Courses

Successful interprofessional education requires all students to learn with and from one another and learning transfer occurs maximally when students are presented with relevant context in which to apply their knowledge (20). Biomedical Scientists rarely interact directly with patients but are involved in 95% of all clinical pathways (21), whereas other healthcare professionals such as Medics, Pharmacists and Nurses are all patient facing. To ensure that the requirements of both programmes were met, academics from both programmes were actively involved in the design and delivery of the workshop. The patient case studies incorporated in the workshop have created an opportunity for collaborative interprofessional practise, which are exemplar of instances where different professionals need to work together in the clinical setting. Other work has shown that collaborative working of student cohorts in a PBL setting can help to foster a positive attitude towards other professional groups, thus, improving interprofessional relations (1).

### Delivering Inter-professional Education Online

The workshop was designed to address a number of different topics and ensured that the content of the three activities were familiar to both programmes but required students to work together across disciplines in order to complete the answers. The activity length was based upon previous work reporting that the average adult has a maximum attention span of 20 min (22). Over three-quarters of students (78.5%) across both programmes positively responded to all of the statements and successfully met the learning outcomes of the IPL workshop. Survey results showed that 88.5% of students agreed that the workshop enabled them to increase their understanding of their own roles within healthcare and their limitations. Furthermore, 90% of the cohort agreed that the workshop increased their understanding the roles of other healthcare professionals involved in patient care; this demonstrates improved awareness of professional roles and responsibilities within the multi-professional team. This is supported by the findings from the thematic analysis which also provide evidence that the tasks set in each of the activities allowed both cohorts to actively disseminate their discipline specific information in the workshop, as well as learn from the other cohort. Previous work has also reported that interprofessional PBL inculcates an appreciation and knowledge of other roles (1, 23, 24). Using the PBL approach to deliver interprofessional education has created an environment for cohorts of students from different backgrounds to listen to others perspectives related to their roles and construct knowledge in collaboration with one another (25, 26).

Students demonstrated knowledge gain, where 74.3% agreed that working with students from a different programme provided an alternative perspective to the case. This was particularly evident for the Medical student cohort, who reported a better understanding of the role of Biomedical Scientists post-workshop, which was identified as being statistically significant (statement nine). All students (100%) agreed that patient's will ultimately benefit from different healthcare professions working together. The activities also provided opportunities for students to identify similarities and differences across their programmes and their practise. There was a common appreciation from both cohorts of students that they each play an essential role in providing high quality care to patients, from the moment a patient first presents to a doctor, to the reporting of test results by the laboratory and collaboration being a key feature. Both the cohorts recognised the need for a multi-disciplinary approach and effective communication in treating patients.

To make the workshop relevant to both programmes and the current climate, Covid-19 clinical presentation and diagnosis was included as part of the tutor-led material. Student cohorts were taught new information about diagnostic tests used to screen for Covid-19. Less than half the student cohort were able to correctly identify diagnostic tests for screening coronavirus infection. Despite the principle behind testing being the same as those used to detect other viral infections, students struggled to apply this knowledge from prior learning, which re-enforces the need for further opportunities to apply pre-existing knowledge.

From the survey students had reported that the online delivery of the IPL enabled them to meet the learning outcomes of the session (78.5%). There was high engagement in the chat function in feedback post activities and the online polling. In face-to-face large group teaching, multiple sessions would be delivered due to cohort size and space requirements, as well as interaction with individual student groups would require multiple staff to monitor and facilitate group discussion, meaning a larger number of staff hours required. In order to participate in interactive polls in traditional IPL, students would have required either specialist equipment or software. Blackboard Collaborate facilitates interactive polling where students can engage anonymously. The interactive polls held during the workshop had high levels of student engagement (with up to 85% of students interacting), which is considerably larger than what is typically observed in a traditional classroom setting. High levels of student attendance and engagement using Blackboard Collaborate has been previously reported, as it also provides the option for students to join sessions wherever they are, through their laptop, computer or smartphone (27). The workshop in the current study included ~200 students across the two cohorts and the online platform successfully accommodated this large number of attendees. Feedback from students showed the online platform enabled interactivity and discussion. Furthermore, other work has reported that virtual interprofessional education is as effective as face-to-face delivery, with students reporting an increase in interprofessional knowledge, attitudes and team-playing attributes (15).

The success of the IPL in part may be explained by the workshop being delivered to only two disciplines. Other work involving IPL activities across five or more healthcare disciplines reported that the results were hindered by excessive disciplines working on a single activity. It has been suggested that reducing the number of disciplines within each team would increase student engagement and improve the interprofessional experience (4, 28). Furthermore, the success of IPL is also dependent upon the relevance of the topics being included to the programmes involved. Despite the benefits of online delivery, there still remains the challenge of timetabling multiple cohorts of students to attend the same session.

## Limitations

Whilst IPL has an important role to play in higher education, the planning and design of the activities is time consuming and resource intensive. The complexity of the planning is increased by academics from across disciplines being involved in the co-creation of the workshop. A previous study also reported that interprofessional education requires three times the amount of preparation compared to traditional course content delivery (29). In the current study we were unable to distinguish the polling responses of Biomedical Science students and Medical students. An additional limitation of hosting online sessions is poor internet connectivity, with a small number of students in the workshop periodically having to re-join the session (exemplified in the table 2 response rate). This is not only frustrating for the student, but also interferes with group discussions and completion of the tasks. Thirty four percent of participants completed the survey and we believe this is reasonable as no incentives were offered to participants. Survey return is difficult in the HE setting, especially as students are encouraged to fill in numerous surveys per module and course and it is not clear if the student sample is biassed relative to the whole cohort. Perhaps if survey return was marked compulsory or incentives were offered it would have increased the survey return rate.

## Future Work

IPL is recognised to be valuable for multi-disciplinary team work and ultimately patient care. Studies suggest embedding IPL early into the curriculum is optimal (30, 31) using relevant and mutually beneficial topics for all undergraduate programmes involved. Previous studies have shown the benefits of IPL, with it helping students greater understanding their own professional role, as well as understand the role of other health professionals (1, 32, 33). To address the limitation of being unable to differentiate polling responses by student group, future work should seek to use an external polling software. This would enable a comparison of student understanding across different cohorts, although the survey data did enable a summative exploration of what student's had learned. Future work could explore the effects of social, cultural and economic backgrounds on the impact of IPL (34) and then seek to address these factors as part of the design of interprofessional education. To further explore any potential benefit of IPL on practise, a longitudinal study investigating the impact of IPE on Biomedical Science graduates who decide to practise as registered Biomedical Scientists within the NHS would be useful to examine the impact of IPE in HE on clinical practise.

## Conclusion

IPL online using a PBL approach was successful in enabling students across two programmes to work together and learn from each other. There was a significant positive impact on the understanding of other healthcare professional roles, markedly for the medical students understanding the Biomedical scientist role in patient care. Online delivery has clear benefits for enabling large cohorts to work together without the limitation of room size and without requiring multiple parallel sessions to deliver events; thus, reducing the overall resource of staff number required for delivery. The online platform successfully facilitated the delivery of one scheduled session for a large number of students, reducing the number of staff required to facilitate polling, breakout groups and discussion. Engagement was evident from the use of the various tools within the online platform, particularly polling where anonymous responses were collated to test student's immediate understanding, whilst respecting the individual learner. With professional bodies recognising the importance of interprofessional teams within the clinical setting, more educators need to collaborate and create IPL opportunities within the curriculum to teach students the true benefits of multi-disciplinary team working, whether online or face to face.

## Data Availability Statement

The raw data supporting the conclusions of this article will be made available by the authors, without undue reservation.

## Ethics Statement

The studies involving human participants were reviewed and approved by Life and Health Sciences Research Ethics Committee (Project #1494). The patients/participants provided their written informed consent to participate in this study.

## Author Contributions

AB contributed to the conception of the study. WL and AB designed the research approach for this study. AB led the design of the online workshop, AB and RP created the Biomedical Science taught material and the workbook students completed. ED created the taught Medical content. All authors contributed to the design of the interactive activities and RP created the interactive polling questions. WL gained ethical approval on project Interdisciplinary Education at Aston and created the online survey. AB and RP analysed and interpreted the data and wrote the first draught of the manuscript. WL and ED provided feedback and contributed to some sections. All authors edited the manuscript, read, and approved the submitted version.

## Conflict of Interest

The authors declare that the research was conducted in the absence of any commercial or financial relationships that could be construed as a potential conflict of interest.

## Publisher's Note

All claims expressed in this article are solely those of the authors and do not necessarily represent those of their affiliated organizations, or those of the publisher, the editors and the reviewers. Any product that may be evaluated in this article, or claim that may be made by its manufacturer, is not guaranteed or endorsed by the publisher.
