# Supplementary material for: A Virtual Approach to Promote Inter-Professional Learning (IPL) Between Biomedical Science and Medicine in Higher Education for the Benefit of Patient Care
Source: Front Public Health. 2021 Oct 6;9:747751. doi: 10.3389/fpubh.2021.747751 (PMC8526844; doi:10.3389/fpubh.2021.747751)
Supplement: Supplementary file 1 [file Data_Sheet_1.pdf]

Online surveys

IPL PRACTICAL LAB FEEDBACK (copy)

Showing 0 of 0 responses

Showing **all** responses

Showing **all** questions

1 Which course are you enrolled on?

Biomedical Science	0
Medicine	0

2 Please select one answer for each row

2.1 I enjoyed the practical session

2.1.a I enjoyed the practical session

Strongly agree	0
Agree	0
Disagree	0
Strongly disagree	0

2.2 The practical session increased my laboratory confidence

2.2.a The practical session increased my laboratory confidence

Strongly agree	0
Agree	0
Disagree	0
Strongly disagree	0

2.3 I feel my laboratary skills have improved

2.3.a I feel my laboratary skills have improved

Strongly agree	**0**
Agree	**0**
Disagree	**0**
Strongly disagree	**0**

3 What did you learn from the IPL practical session?

No responses

3.a How could the practical session be improved?

No responses
